# Remimazolam *vs.* propofol for general anaesthesia in elderly patients: a meta-analysis with trial sequential analysis

**DOI:** 10.1097/EJA.0000000000002042

**Published:** 2024-08-12

**Authors:** Eduardo Maia Pereira, Vitor Ryuiti Moraes, Mariana Gaya da Costa, Tatiana Souza do Nascimento, Eric Slawka, Carlos Galhardo Júnior, Michel MRF Struys

**Affiliations:** From the Federal University of Minas Gerais, Department of Medicine, Belo Horizonte, Brazil (EMP), Evangelical University of Goias, Department of Medicine, Anápolis, Brazil (VRM), University of Groningen and University Medical Center of Groningen, Department of Anaesthesiology, Groningen, The Netherlands (MGdC, MMRFS), Cardoso Fontes Federal Hospital, Department of Anaesthesiology (TSdN), Federal University of Rio de Janeiro, Department of Medicine, Rio de Janeiro, Brazil (ES), McMaster University & DeGroote School of Medicine, Department of Anaesthesiology, Hamilton, Ontario, Canada (CGJ), and Department of Basic and Applied Medical Sciences, Ghent University, Ghent, Belgium (MMRFS)

## Abstract

**BACKGROUND:**

Elderly patients comprise an increasing proportion of patients undergoing surgery, and they require special attention due to age-related physiological changes. Propofol is the traditional agent for anaesthesia, and recently, remimazolam, a novel ultra-short-acting benzodiazepine, has emerged as an alternative to propofol in general anaesthesia.

**OBJECTIVES:**

We aim to compare remimazolam *vs*. propofol for general anaesthesia in elderly patients regarding hypotension, induction characteristics, haemodynamics and recovery outcomes.

**DESIGN:**

Meta-analysis with sensitivity and trial sequential analyses (TSA) to assess inconsistencies. Risk ratios and mean differences with 95% confidence intervals (95% CIs) were computed using a random effects model. Subgroups and meta-regression according to anaesthesia methods were also performed.

**DATA SOURCES:**

We systematically searched MEDLINE, Embase and Cochrane for randomised controlled trials (RCTs) up to January 1, 2024.

**ELIGIBILITY CRITERIA:**

Patients at least 60 years old, comparing remimazolam *vs*. propofol for general anaesthesia.

**RESULTS:**

Eleven RCTs (947 patients) were included. Compared with propofol, remimazolam was associated with lower postinduction and intra-operative hypotension (RR 0.41, 95% CI 0.27 to 0.62, *P* < 0.001) and incidence of bradycardia (risk ratio 0.58, 95% CI 0.34 to 0.98, *P* = 0.04), with a higher heart rate (*P* = 0.01). The incidence of injection pain was lower (*P* < 0.001), but remimazolam was associated with a longer time to loss of consciousness (*P* < 0.001) and a higher bispectral index at loss of consciousness (*P* = 0.04). No differences were found for mean arterial pressure, emergence time, extubation time and incidence of emergence agitation. The TSA was consistent and achieved the required information size for hypotension.

**CONCLUSIONS:**

Remimazolam significantly reduced the risk of hypotension, bradycardia and injection pain, despite an increase in the time to loss of consciousness. Remimazolam appears to be an effective and well tolerated alternative to propofol in elderly patients undergoing general anaesthesia.

This article accompanies the editorial: Hansen, Tom G.; Van de Velde, Marc. Enhancing anaesthetic care for the elderly. Propofol versus remimazolam for general anaesthesia. *Eur J Anaesthesiol* 2024; **41**:627-628.

**KEY POINTS**

- First meta-analysis to assess remimazolam for general anaesthesia in the elderly.
- Compared with propofol, remimazolam decreased the risk of hypotension and bradycardia.
- Time to loss of consciousness was higher with remimazolam with a higher bispectral index.
- No differences in the anaesthetic recovery characteristics were found.

## Introduction

Many studies have found that peri-operative hypotension is associated with various adverse events, such as myocardial infarction, death and increased hospital costs.^1,2^ Currently, elderly patients represent an increasing proportion of the surgical population and are more vulnerable to peri-operative complications, especially hypotension.^3^ Previous findings suggest that, in this population, both postinduction and intra-operative hypotension seem to be associated with poor outcomes, including increased mortality.^4^

Propofol, a sedative-hypnotic, is one of the most frequently used intravenous anaesthetics to induce and maintain general anaesthesia, given its rapid onset and smooth recovery.^5^ However, it is associated with side effects such as bradycardia, injection pain and hypotension, particularly in the geriatric population.^6,7^ Remimazolam, a novel short-acting benzodiazepine, targets the gamma-aminobutyric acid A (GABA-A) receptors and is rapidly converted to inactive metabolites by carboxylesterase 1.^8^ In procedural sedation, it shows better haemodynamic profiles and lower incidence of side effects compared with other sedatives.^9^ Furthermore, remimazolam sedative effects can be reversed by flumazenil, favouring its safety for anaesthetic procedures.

Previous studies have assessed the use of remimazolam in procedural sedation and general anaesthesia. Recent studies involving adult patients indicate that, compared with propofol, remimazolam shows a lower risk of intra-operative hypotension and a comparable recovery period.^10^ However, no meta-analysis has compared these two drugs, which have different pharmacokinetic and pharmacodynamic profiles with regard to general anaesthesia for elderly patients, which is an important consideration for peri-operative anaesthetic management.^11^ Therefore, we performed a systematic review and meta-analysis comparing propofol *vs*. remimazolam for general anaesthesia in elderly patients undergoing surgery, in terms of efficacy and safety during the peri-operative period.

## Material and methods

This study was conducted and reported based on the Preferred Reporting Items for Systematic Reviews and Meta-analyses (PRISMA) and the Cochrane guidelines.^12,13^ The protocol was registered in the International Prospective Register of Systematic Reviews (PROSPERO), identifier CRD42023495765, on 31 December 2023.

### Eligibility criteria

The inclusion criteria were randomised controlled trials (RCTs); including elderly patients (≥60 years old) only; comparing propofol with remimazolam during the induction and/or maintenance of anaesthesia, and reporting at least one of the outcomes of interest. Exclusion criteria were trial protocols, abstracts only, studies published in any language other than English, and application of regional anaesthesia or sedation only.

### Outcome definitions

The primary outcome was the overall incidence of hypotension during the intra-operative period (i.e. during anaesthesia induction and/or maintenance). Secondary outcomes included; characteristics of the induction, i.e., time to loss of consciousness (LOC), incidence of injection pain and anaesthetic depth; haemodynamics, i.e., mean arterial pressure (MAP), heart rate (HR) and incidence of bradycardia; and recovery (incidence of emergence agitation, emergence time and extubation time). Given that all studies were randomised and baseline characteristics were comparable, we collected the lowest mean value in each group for MAP and HR after the beginning of drug administration and computed the mean difference, according to the Cochrane guidelines.^13^ The anaesthetic depth was defined as the bispectral index (BIS), and the time point for data collection was set at the moment of loss of consciousness. If several doses of remimazolam were reported, we only analysed the data of groups with the lower dose, as previous dose-response analyses recommended lower dosages for elderly patients.^14–16^

### Study selection and data extraction

Eligible studies were searched on MEDLINE, Embase and Cochrane. The final search was performed on 1 January 2024, with no restrictions on publication year, country of origin or journal. The complete search strategy is listed in Supplemental Digital Content (SDC) 1 (Table A.1). Two independent reviewers (EM and MG) selected eligible trials according to the inclusion and exclusion criteria and performed a cross-section. After removing the duplicates, all results were pooled and the screening by title and abstract was performed. Finally, the remaining articles were read in full. Disagreements between the two reviewers were resolved through a discussion with a third reviewer (CG). When continuous data were reported as median and interquartile range, the values were converted to mean and standard deviation using Wan's method,^17^ and values reported in graphs were collected with the PlotDigitalizer (https://plotdigitizer.com) software.

### Risk of bias and certainty of evidence

Two reviewers (EM and VR) independently assessed the risk of bias with the revised Cochrane risk of bias tool for randomised trial 2 (RoB 2).^18^ The Robvis tool was used to create the final figure.^19^ The level of certainty of the evidence was assessed with the Grading of Recommendations Assessment, Development and Evaluation (GRADE) system.^20^ The same reviewers assessed the outcomes, and any disagreements were resolved through discussion among all authors. The final figure was created with the GRADEpro software (gradepro.org).^21^

### Statistical analysis

Data analysis was performed with the Review Manager 5.4 (Cochrane Collaboration, 2020, Copenhagen, Denmark). Risk ratios and mean differences with 95% CIs were applied for dichotomous and continuous outcomes, respectively. The random-effects model was chosen for all analyses due to anticipated heterogeneity. Subgroup analyses were performed according to timing of hypotension (i.e. postinduction or intra-operative hypotension), flumazenil use for recovery outcomes and technique of remimazolam and propofol use (for induction only or for both induction and maintenance of anaesthesia). Statistical significance was set at *P* value less than 0.05.

Heterogeneity was assessed with the Cochran Q and *I*^*2*^ statistics and categorised as low (*I*^*2*^ = 0 to 40%), moderate (*I*^*2*^ = 30 to 60%), substantial (*I*^*2*^ = 50 to 90%) or considerable (*I*^*2*^ = 75 to 100%), according to the Cochrane guidelines.^13^ Publication bias was investigated by funnel plots and Egger's test. A sensitivity analysis by omitting each study individually was performed under the random-effects model. We also performed a meta-regression to assess the impact of flumazenil use on recovery outcomes. The R software was used for these analyses.^22^

Furthermore, we performed a trial sequential analysis (TSA) to estimate the required information size and assess the risk of type I and II errors. The thresholds for the *Z* score were set using the O’Brien-Fleming alpha spending function, and a random-effects model was applied (DerSimonian-Laird method). A type I error of 0.05 and a type II error of both 0.10 and 0.01 (power = 90 and 99%, respectively) were allowed. We used the software TSA 0.9 for the analysis.^23^

## Results

### Study selection and characteristics

**Fig. 1:**
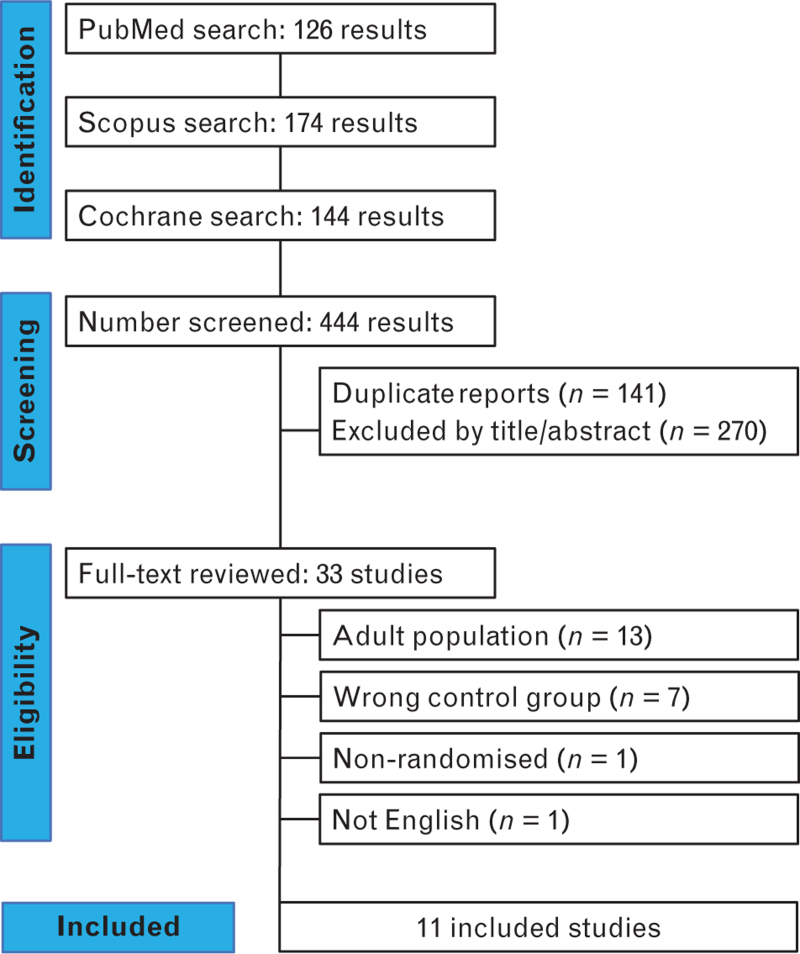
PRISMA flow diagram of study screening and selection.

Study selection is summarised in Fig. 1. A total of 444 articles were initially identified. Following the exclusion of 141 duplicates and 270 articles by abstract screening, 33 articles underwent full-text review. Finally, 11 trials were included,^24–34^ totalling 947 patients; 472 (49.8%) were assigned to remimazolam and 475 (50.2%) to propofol groups. Flumazenil was used in four trials,^24,28–30^ and remimazolam was given for both induction and maintenance of general anaesthesia in nine studies.^24–32^Table 1 summarises baseline characteristics of included studies.

**Table 1:** Baseline characteristics of included randomised controlled trials

Ref.	Country	Patients, *n* Remi vs. Pro	Age (years) Remi vs. Pro	ASA status	Surgery	Flumazenil	Induction dose Remi vs. Pro	Maintenance dose Remi vs. Pro
Zhang *et al*.^24^	China	30 vs. 29	74.3 ± 10.6 vs. 75.0 ± 9.9 ^a^	II - III	Hip arthroplasty	Yes	0.2 to 0.4 mg kg^–1^ vs. 1.5 to 2 mg kg^–1^	0.3 to 0.5 mg kg^–1^ h^–1^ vs. 4 to 8 mg kg^–1^ h^–1^
Yang *et al*.^25^	China	147 vs. 153	68 (65 to 71) vs. 68 (65 to 71) ^b^	I - III	Mixed orthopaedics	No	0.2 to 0.3 mg kg^–1^ vs. 1 to 1.5 mg kg^–1^	According to BIS
Duan *et al*.^26^	China	30 vs. 30	67.8 ± 3.2 vs. 68.7 ± 2.9 ^a^	I - III	Hip arthroplasty	No	0.2 to 0.4 mg kg^–1^ vs. 1.5 to 2 mg kg^–1^	0.3 to 0.5 mg kg^–1^ h^–1^ vs. 4 to 8 mg kg^–1^ h^–1^
Kuang *et al*.^27^	China	42 vs. 42	65.4 ± 3.9 vs. 65.2 ± 4.4 ^a^	I - III	Lobectomy	No	0.3 mg kg^–1^ vs. 2 mg kg^–1^	0.6 to 1.2 mg kg^–1^ h^–1^ vs. 2 to 10 mg kg^–1^ h^–1^
So *et al*.^28^	South Korea	42 vs. 39	74.5 (70 to 78.3) vs. 76 (70 to 81) ^b^	I - III	Cholecystectomy	Yes	6 mg kg^–1^ vs. 1 to 1.5 mg kg^–1^	1 to 2 mg kg^–1^ h^–1^ vs. 100 μg kg^–1^ ml^–1^
Toyota *et al*.^29^	Japan	20 vs. 19	80 (79 to 83) vs. 81 (79 to 82) ^b^	II - III	Spine	Yes	12 mg kg^–1^ h^–1^ vs. 3 μg ml^–1^	According to BIS
Jeon *et al*.^30^	South Korea	60 vs. 62	70.9 ± 4.3 vs. 71.5 ± 4.3 ^a^	I - III	Cholecystectomy TURBT	Yes	6 mg kg^–1^ h^–1^ vs. 4 μg ml^–1^	1 - 2 mg kg^–1^ h^–1^ vs. 2.5 - 4 μg mL^–1^
He *et al*.^31^	China	28 vs. 29	70.3 ± 4.1 vs. 70.8 ± 3.5 ^a^	II - III	Mixed Transurethral	No	6 mg kg^–1^ h^–1^ vs. 60 mg kg^–1^ h^–1^	0.5 - 2 mg kg^–1^ h^–1^ vs. 4 to 10 mg kg^–1^ h^–1^
Kim *et al*.^32^	South Korea	23 vs. 22	73 (65 to 86) vs. 68 (65 to 82) ^b^	I - II	Mixed	No	6 mg kg^–1^ h^–1^ vs. 4 μg ml^–1^	0.8 to 1.2 mg kg^–1^ h^–1^ vs. 2.5 to 3 μg ml^–1^
Xu *et al*.^33^	China	30 vs. 30	69.9 ± 4.3 vs. 68.6 ± 3.3 ^a^	I - II	Lower limbs	No	0.2 mg kg^–1^ vs. 1.5 mg kg^–1^	-
Gao *et al*.^34^	China	20 vs. 20	67.2 ± 4.4 vs. 67.2 ± 4.4 ^a^	II - III	Carotid endarterectomy	No	0.3 mg kg^–1^ vs. 1.5 to 2 mg kg^–1^	-

ASA, American Society of Anesthesiology; BIS, bispectral index; Pro, propofol; Remi, remimazolam; TURBT, transurethral resection of bladder tumour.

a Mean ± standard deviation.

b Median (interquartile range).

The risk of bias assessment is shown in SDC 2 (Fig. B.1). The overall risk of bias was classified as ‘some concerns’ in four studies^24,28,29,33^ and ‘low’ in the remaining seven studies.^25–27,30–32,34^ The GRADE summary of findings is shown in SDC 1 (Table A.2). The certainty of the evidence for the primary outcome of hypotension was considered high. However, although the risk of bias assessment indicates that the overall quality of included studies was reasonable, the GRADE assessment showed a considerably low certainty of the evidence for some outcomes, such as anaesthetic depth and emergence agitation, primarily due to the high heterogeneity, a limited number of studies and wide CIs.

### Haemodynamics

**Fig. 2:**
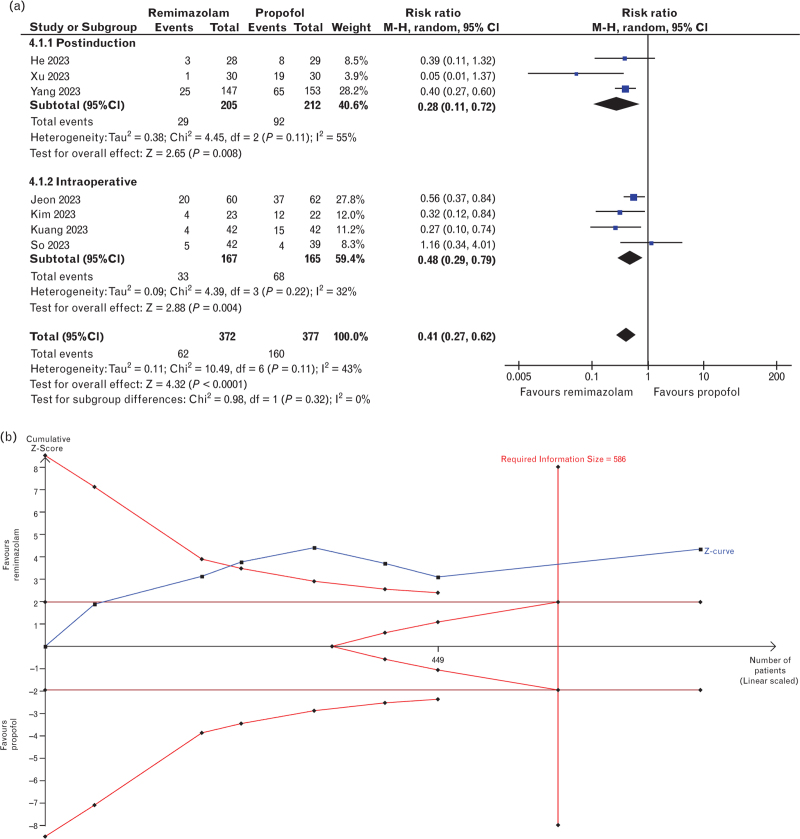
Forest plot and trial sequential analysis for the incidence of hypotension.

**Fig. 3:**
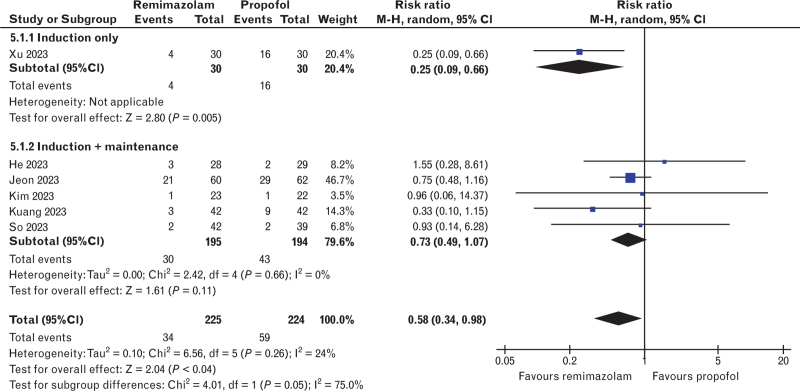
Forest plot for the incidence of bradycardia. Remimazolam reduced the incidence of bradycardia when compared with propofol.

**Fig. 4:**
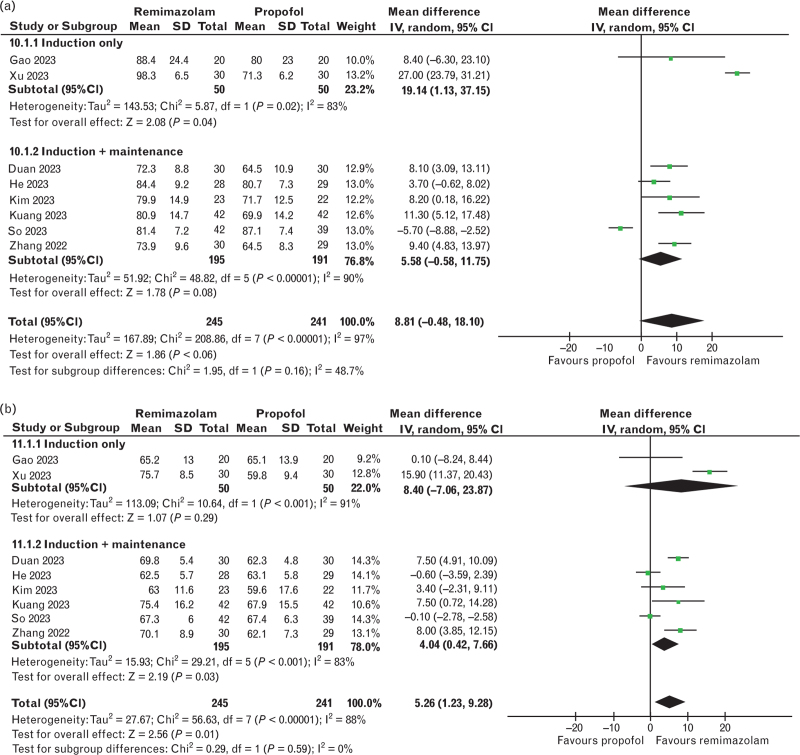
Forest plots for the mean arterial pressure and heart rate.

The incidence of hypotension was reported in seven trials,^25,27,28,30–33^ totalling 749 patients. The results showed a lower risk of hypotension with remimazolam use compared with propofol (rosk ratio 0.41, 95% CI 0.27 to 0.62, *P* < 0.001, *I*^*2*^ = 43%, Fig. 2a). Similarly, remimazolam correlated with a lower risk of intra-operative bradycardia (risk ratio 0.58, 95% CI 0.34 to 0.98, *P* = 0.04, *I*^*2*^ = 24%, six studies,^27,28,30–33^ 449 patients, Fig. 3). The MAP and HR were reported in eight studies,^24,26–28,31–34^ totalling 486 patients, and no difference was found between both groups for the MAP (mean difference 8.81 mmHg, 95% CI -0.48 to 18.10, *P* = 0.06, *I*^*2*^ = 97%), but the HR was higher in the remimazolam group (mean difference 5.26 bpm, 95% CI 1.23 to 9.28, *P* = 0.01, *I*^*2*^ = 88%) (Fig. 4a, 4b).

(a) Remimazolam significantly decreased the risk of postinduction, intra-operative and overall hypotension compared with propofol. (b) Trial sequential analysis on hypotension crossed the trial sequential monitoring boundary and achieved the required information size (power=99%).

(a) There was no difference in the mean arterial pressure between groups. (b) Remimazolam showed a lower heart rate compared with propofol.

### Induction and recovery

**Fig. 5:**
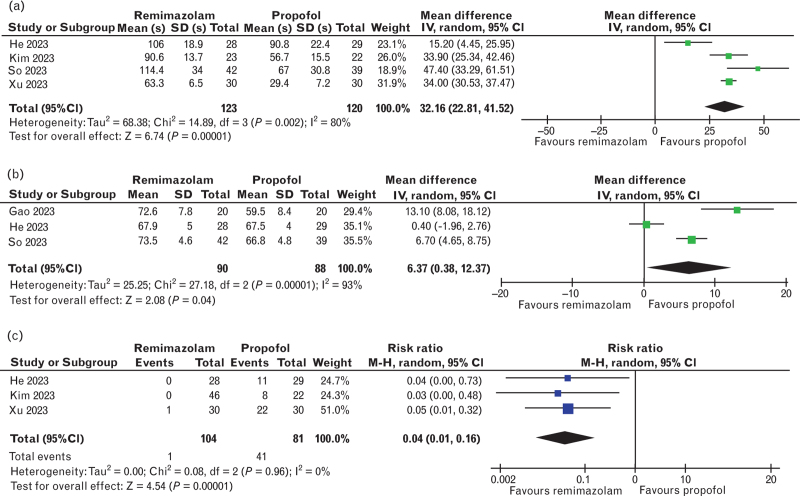
Forest plots for the induction characteristics.

The assessment of induction characteristics is shown in Fig. 5. The time to LOC was analysed by four studies (243 patients)^28,31–33^ and was higher in the remimazolam group (mean difference 32.16 s, 95% CI 22.8 to 41.5, *P* < 0.001, *I*^*2*^ = 80%) (Fig. 5a). Similarly, at the moment of LOC, the BIS values, reported in three studies^28,31,34^ (178 patients), were higher in the remimazolam group (mean difference 6.37, 95% CI 0.38 to 12.37, *P* < 0.001, *I*^*2*^ = 93%), (Fig. 5b). In contrast, remimazolam was associated with a lower risk of injection pain (risk ratio 0.04, 95% CI 0.01 to 0.16, *P* < 0.001, *I*^*2*^ = 0%) (three studies,^31–33^ 185 patients, Fig. 5c). Regarding the recovery period, the results are shown in the supplemental content (SDC 2, Fig. B.2). No significant differences were found in emergence time (mean difference -0.11 min, 95% CI -1.05 to 0.83, *P* = 0.82, *I*^*2*^ = 77%, six studies,^24,26,28,29,32,34^ 324 patients), extubation time (mean difference 0.40 min, 95% CI -0.92 to 1.73, *P* = 0.55, *I*^*2*^ = 89%, seven studies,^24–26,28–30,32^ 706 patients) and incidence of emergence agitation (risk ratio 0.64, 95% CI 0.17 to 2.42, *P* = 0.51, *I*^*2*^ = 70%, three studies,^24–26^ 419 patients).

(a) Remimazolam was associated with a longer time to loss of consciousness. (b) Remimazolam was associated with a higher bispectral index. (c) Remimazolam was associated with a lower incidence of injection pain.

### Subgroup analysis

The results of the incidence of hypotension were consistent among studies assessing postinduction and intra-operative hypotension (Fig. 2a). The subgroup analysis based on administration technique (i.e. induction only or induction and maintenance) is shown in all forest plots for haemodynamic and recovery outcomes (Figs. 3 and 4 + Fig. B.2). The analysis was limited given that only two studies applying drugs solely for induction were included in any outcome. Nonetheless, the results were consistent among subgroups for the emergence time; however, there were differences between subgroups and the overall result in the analysis of bradycardia, MAP and HR. Furthermore, the analysis could not be performed for extubation time and emergence agitation, as no studies assessing only the induction included these outcomes.

Assessment according to flumazenil use is shown in the supplemental content (SDC 2). Subgroup analysis of studies that used flumazenil remained statistically insignificant for the emergence and extubation times (Fig. B.3). The emergence agitation could not be assessed due to an insufficient number of studies. Meta-regression analysis for the recovery outcomes based on flumazenil use was statistically insignificant for emergence and extubation times, despite the regression coefficient favouring flumazenil use (Fig. B.4). The analysis of emergence agitation could not be performed due to a small number of studies.

### Heterogeneity and sensitivity analysis

The heterogeneity and sensitivity analyses are shown in the supplemental content (SDC 2). For the primary outcome of hypotension, the sensitivity analysis showed that the exclusion of any study would not change the effect size, and the heterogeneity is mostly attributed to one study.^33^ Likewise, the analysis was consistent for the time to LOC, injection pain, HR, emergence time and extubation time. The removal of one particular study in the assessment of anaesthetic depth,^31^ MAP^28^ and emergence agitation^25^ would also change the pooled effect size. For bradycardia, the removal of four studies^27,28,32,33^ makes the results statistically insignificant (Fig. B.5).

The Egger's test was not significant for publication bias in any of the outcomes (SDC 1, Table A.3). Similarly, qualitative analysis of funnel plots did not show considerable asymmetries (SDC 2, Fig. B.6). However, publication bias could not be assessed adequately due to the small number of studies in each outcome. Inspection of funnel plots has limited value with small sample sizes, and according to the Cochrane guidelines,^13^ Egger's test does not offer consistent results with less than 10 studies.

### TSA

The TSA for the primary outcome of hypotension crossed the monitoring boundary and achieved the required information size (Fig. 2b, power = 99%). With powers of both 90 and 99%, the analysis of bradycardia, anaesthetic depth and MAP did not cross the monitoring and the required information size boundaries, and the graph of HR crossed the former, but not the latter (SDC 2, Fig. B.8 and B.9). The outcomes of time to LOC, incidence of injection pain, emergence time, extubation time and emergence agitation could not be assessed with TSA, as the results were not renderable due to little information.

## Discussion

In this meta-analysis of general anaesthesia of elderly patients, comparing remimazolam with propofol, remimazolam was associated with a lower risk of hypotension and bradycardia; greater HR despite no difference in MAP; longer time to LOC and less profound anaesthetic depth, as observed by higher BIS values; and lower incidence of injection pain, with no significant differences in recovery outcomes. To our knowledge, this is the first meta-analysis assessing remimazolam's applicability specifically in elderly patients. Older patients have an increased risk of perioperative morbidity and mortality and therefore require specific considerations.^35^ Age-related physiological changes and previous comorbidities commonly found in these patients lead to particular peri-operative characteristics, such as increased sensitivity to anaesthetics and longer recovery time from anaesthesia.^11^ Propofol, for example, achieves the same effect in elderly patients with only 50 to 70% of the dose required for younger patients.^36^ To date, the majority of the evidence regarding remimazolam has centred on procedural sedation in adult patients. In a meta-analysis assessing adult patients,^9^ the authors found that remimazolam-based sedation for endoscopies showed a lower risk of hypotension and bradycardia, with similar recovery and discharge characteristics. However, given the recent approval for the use of remimazolam in general anaesthesia in various countries,^37^ the above-mentioned metabolic particularities in geriatric patients must be clarified.

Hypotension is one of the most common adverse events reported during general anaesthesia, and its risk increases with age when propofol is administered.^7^ In elderly patients, peri-operative hypotension may compromise cognitive performance and increase the risk of mortality, which warrants the use of alternative agents.^38^ In our study, we found that remimazolam was associated with a lower incidence of both postinduction and intra-operative hypotension, and the results were robust to sensitivity analysis and TSA. This aligns with prior studies involving adult patients,^10,39^ indicating that remimazolam can be an effective alternative to propofol for reducing the risk of hypotension regardless of age. Similarly, the incidence of bradycardia was lower in the remimazolam group, with a significant reduction in the HR in the propofol group. This was also found in a previous meta-analysis that compared both drugs for induction of general anaesthesia in adults.^40^ Propofol is thought to induce bradycardia through different mechanisms, such as changes in action potential amplitude and duration, leading to an increased risk of arrhythmias including asystole.^41,42^ Given that elderly patients seem to be more susceptible to bradycardia under general anaesthesia,^43^ remimazolam may also be an effective alternative to mitigate possible cardiac-related adverse events.

We also found that remimazolam was associated with a longer time to LOC and higher BIS values at the moment of LOC. Our results are consistent with a prior meta-analysis of remimazolam *vs*. propofol for general anaesthesia in adults.^10^ Their study was the only previous meta-analysis that compared the BIS between both drugs for general anaesthesia and also faced substantial heterogeneity. However, the authors also found a comparable efficacy of anaesthesia induction, a comparison we could not perform due to lack of information. In contrast, the longer time to LOC in our study differs from the results of the meta-analysis by Chang *et al.*,^9^ who found no difference in this outcome in adult patients undergoing procedural sedation. This is probably due to the additional drugs administered for anaesthesia induction; nonetheless, several studies have been conducted to estimate the appropriate single-bolus induction dose of remimazolam for elderly patients. In a time-to-event model trial, Chae *et al.*^15^ suggested a dose of 0.14 to 0.19 mg kg^–1^ for patients aged 60 to 80 years, whereas Oh *et al.*^16^ proposed a 0.25 mg kg^–1^ dose to better achieve loss of consciousness. In our study, different doses and administration methods between trials might have contributed to the high heterogeneity found for the time to LOC; in addition, the application of the BIS algorithm calibration, as well as other electroencephalogram derivatives, to the effects of benzodiazepines remains controversial, which precludes its assessment for remimazolam use.^37^ The relatively small sample limited our analysis of induction outcomes; nonetheless, the sensitivity analyses were consistent for both outcomes, but not for the anaesthetic depth, which also failed to achieve definitive conclusions in the TSA.

In addition, we found that the recovery characteristics were similar among both groups. Regarding the emergence time and extubation time, our findings were robust to sensitivity analysis and similar to the results in the meta-analysis by Ko *et al.*^10^ in adult patients, indicating the consistency of the results. However, our assessment of emergence agitation only comprised three studies,^24–26^ and no difference was found between groups. Among these, Duan *et al.*^26^ found a significant reduction in the incidence of emergence agitation in the remimazolam group, while Yang *et al.*^25^ found a lower incidence of this event in the propofol group, despite the results being statistically insignificant. Very few studies included the cognitive changes associated with the use of remimazolam, and to our knowledge, no previous meta-analysis has assessed this particular outcome in elderly patients. In our study, we could not analyse the incidence of delirium due to the small number of trials, although there are ongoing trial protocols focused on this endpoint in elderly patients.^44,45^ In older patients, the assessment of postoperative cognitive changes with remimazolam is particularly important, given that benzodiazepines are associated with the development of delirium and emergence agitation in this population.^46^ Given the limited number of trials, additional studies are required to provide more evidence in this context.

Furthermore, as few studies administered flumazenil, the analysis of remimazolam reversal was limited in this study, as well as the subgroup analysis and meta-regression test. We did not find any statistically significant change in postoperative endpoints according to flumazenil administration. In a recent meta-analysis involving adult patients under general anaesthesia receiving propofol or remimazolam with flumazenil, Wu *et al*.^47^ found a lower emergence time and extubation time in the remimazolam/flumazenil group. As sedative reversal is a particular advantage of benzodiazepines, more studies assessing the impact of flumazenil on remimazolam anaesthesia would be of great importance.

Our study has some limitations. Despite our efforts to address the primary sources of heterogeneity among studies through different methods, the limited number of studies with common characteristics precludes a more granular assessment of the results. The differences in dosages and anaesthetic strategies might have contributed to the heterogeneity. Likewise, different types of surgery might also be a source of heterogeneity. Due to the limited sample of studies, we could not perform more in-depth analyses, such as subgroups for dosage and type of surgery (i.e. orthopaedic *vs*. nonorthopaedic). This lack of information also restricted the analyses of subgroups, Egger's test, funnel plots and meta-regression. However, the majority of endpoints were robust to sensitivity analysis, which indicates the consistency of our results. Furthermore, although high for the primary outcome of hypotension, the GRADE assessment for the remaining outcomes was overall low, mostly due to low information and high heterogeneity. In addition, 10 of the 11 studies were single-centre trials and all were conducted in Asian countries (China, South Korea and Japan), and similarity between ethnic or geographical backgrounds may also limit the findings of our study. Given the relatively small sample for pooled analyses, our findings support that further large-scale trials are required to understand better the impact of remimazolam anaesthesia in elderly patients, especially in terms of dosage, type of surgery, flumazenil use, adverse events and recovery characteristics.

## Conclusion

In elderly patients undergoing surgery with general anaesthesia, the use of remimazolam, compared with propofol, showed a significant reduction in the incidence of hypotension and bradycardia, with no differences in the recovery characteristics, despite a longer time to loss of consciousness and higher BIS values. Remimazolam may be considered an effective and well tolerated alternative to propofol for general anaesthesia in elderly patients.

## Supplementary Material

Supplemental Digital Content

## Supplementary Material

Supplemental Digital Content
