# Supplementary material for: Remimazolam vs. propofol for general anaesthesia in elderly patients: a meta-analysis with trial sequential analysis
Source: Eur J Anaesthesiol. 2024 Aug 12;41(10):738–48. doi: 10.1097/EJA.0000000000002042 (PMC11377052; doi:10.1097/EJA.0000000000002042)
Supplement: Supplemental Digital Content [file ejanet-41-738-s001.docx]

**Article title:** Remimazolam versus propofol for general anaesthesia in elderly patients: a meta-analysis with trial sequential analysis

**Journal:** European Journal of Anaesthesiology

**Authors:** Eduardo Maia Martins Pereira, Vitor Ryuiti Yamamoto Moraes, Mariana Gaya da Costa, Tatiana Souza do Nascimento, Eric Slawka, Carlos Galhardo Júnior, Michel MRF Struys

**Corresponding author:**

**Mariana Gaya da Costa, MD/PhD**

Department of Anaesthesiology, University of Groningen, University Medical Center of Groningen

Hanzeplein 1, 9713 GZ Groningen, The Netherlands

m.gaya.da.costa@umcg.nl

**Supplementary Tables**

**Table A.1.** Search strategy and number of results for each database.

**Table A.2.** Grading of Recommendations Assessment, Development, and Evaluation (GRADE) assessment of the level of certainty of the evidence.

**Table A.3.** Egger’s linear regression test for all outcomes.

**Table A.1.** Search strategy and number of results for each database. The same search strategy was applied to all databases. The final search was performed on January 1, 2024.

| Search strategy: | (remimazolam OR byfavo) AND (elderly OR elder OR older OR senile OR geriatric) |
| --- | --- |
| Database | **Number of results** |
| MEDLINE | **126** |
| Embase | **174** |
| Cochrane | **144** |

**Table A.2.** Grading of Recommendations Assessment, Development, and Evaluation (GRADE) assessment of the level of certainty of the evidence.

| **Remimazolam compared to propofol in elderly patients undergoing surgery with general anaesthesia** | | | | | |
| --- | --- | --- | --- | --- | --- |
| **Patient or population:** elderly patients undergoing surgery with general anaesthesia  **Setting:** operating room  **Intervention:** remimazolam  **Comparison:** propofol | | | | | |
| **Outcomes** | **№ of participants  (studies)  Follow-up** | **Certainty of the evidence  (GRADE)** | **Relative effect  (95% CI)** | **Anticipated absolute effects** | |
|  |  |  |  | **Risk with propofol** | **Risk difference with remimazolam** |
| Hypotension | 749  (7 RCTs) | ⨁⨁⨁⨁  High | **RR 0.41**  (0.27 to 0.62) | 424 per 1.000 | **250 fewer per 1.000**  (310 fewer to 161 fewer) |
| Time to LOC | 243  (4 RCTs) | ⨁⨁◯◯  Low^a,b,c^ | - |  | MD **32.16 seconds higher**  (22.81 higher to 41.52 higher) |
| Injection pain | 185  (3 RCTs) | ⨁⨁⨁⨁  High^c^ | **RR 0.04**  (0.01 to 0.16) | 506 per 1.000 | **486 fewer per 1.000**  (501 fewer to 425 fewer) |
| Anaesthetic depth | 178  (3 RCTs) | ⨁◯◯◯  Very low^b,c,d^ | - |  | MD **6.37 higher**  (0.38 higher to 12.37 higher) |
| MAP | 486  (8 RCTs) | ⨁⨁◯◯  Low^a,b^ | - |  | MD **8.81 mmHg higher**  (0.48 lower to 18.1 higher) |
| HR | 486  (8 RCTs) | ⨁⨁◯◯  Low^a,b^ | - |  | MD **5.26 bpm higher**  (1.23 higher to 9.28 higher) |
| Bradycardia | 449  (6 RCTs) | ⨁⨁⨁◯  Moderate^c^ | **RR 0.58**  (0.34 to 0.98) | 263 per 1.000 | **111 fewer per 1.000**  (174 fewer to 5 fewer) |
| Emergence agitation | 419  (3 RCTs) | ⨁◯◯◯  Very low^b,c,d^ | **RR 0.64**  (0.17 to 2.42) | 108 per 1.000 | **39 fewer per 1.000**  (90 fewer to 154 more) |
| Emergence time | 324  (6 RCTs) | ⨁⨁◯◯  Low^b,d^ | - |  | MD **0.11 minutes lower**  (1.05 lower to 0.83 higher) |
| Extubation time | 706  (7 RCTs) | ⨁⨁◯◯  Low^a,b^ | - |  | MD **0.4 minutes higher**  (0.92 lower to 1.73 higher) |
| ***The risk in the intervention group** (and its 95% confidence interval) is based on the assumed risk in the comparison group and the **relative effect** of the intervention (and its 95% CI).   **CI:** confidence interval; **MD:** mean difference; **RR:** risk ratio | | | | | |
| **GRADE Working Group grades of evidence**  **High certainty:** we are very confident that the true effect lies close to that of the estimate of the effect.  **Moderate certainty:** we are moderately confident in the effect estimate: the true effect is likely to be close to the estimate of the effect, but there is a possibility that it is substantially different.  **Low certainty:** our confidence in the effect estimate is limited: the true effect may be substantially different from the estimate of the effect.  **Very low certainty:** we have very little confidence in the effect estimate: the true effect is likely to be substantially different from the estimate of effect. | | | | | |

#### **Explanations**

a. The proportion of information from trials with concerns about the risk of bias may affect the interpretation of the results.

b. High heterogeneity (I2 > 50%). Downgraded by one level for inconsistency

c. A large number of included studies do not contribute to this outcome. Downgraded by one level for publication bias.

d. Studies include few patients and events and thus have a wide CI. Downgraded by one level for imprecision.

**Table A.3.** Egger’s linear regression test for all outcomes.

| **Outcome** | **Egger’s test** |
| --- | --- |
| Hypotension | Test result: t = -1.02, df = 5, p-value = 0.3548 |
| Time to loss of consciousness | Test result: t = -0.22, df = 2, p-value = 0.8466 |
| Incidence of injection pain | Test result: t = -0.77, df = 1, p-value = 0.5828 |
| Anaesthetic depth | Test result: t = 0.59, df = 1, p-value = 0.6618 |
| Mean arterial pressure | Test result: t = -0.08, df = 6, p-value = 0.9364 |
| Heart rate | Test result: t = 0.61, df = 6, p-value = 0.5662 |
| Bradycardia | Test result: t = -0.20, df = 4, p-value = 0.8545 |
| Incidence of emergence agitation | Test result: t = -1.20, df = 1, p-value = 0.4421 |
| Emergence time | Test result: t = -0.09, df = 4, p-value = 0.9325 |
| Extubation time | Test result: t = 0.75, df = 5, p-value = 0.4866 |
