# Supplementary material for: Remimazolam vs. propofol for general anaesthesia in elderly patients: a meta-analysis with trial sequential analysis
Source: Eur J Anaesthesiol. 2024 Aug 12;41(10):738–48. doi: 10.1097/EJA.0000000000002042 (PMC11377052; doi:10.1097/EJA.0000000000002042)

**Article title:** Remimazolam versus propofol for general anaesthesia in elderly patients: a meta-analysis with trial sequential analysis

**Journal:** European Journal of Anaesthesiology

**Authors:** Eduardo Maia Martins Pereira, Vitor Ryuiti Yamamoto Moraes, Mariana Gaya da Costa, Tatiana Souza do Nascimento, Eric Slawka, Carlos Galhardo Júnior, Michel MRF Struys

**Corresponding author:**

**Mariana Gaya da Costa, MD/PhD**

Department of Anaesthesiology, University of Groningen, University Medical Center of Groningen

Hanzeplein 1, 9713 GZ Groningen, The Netherlands

m.gaya.da.costa@umcg.nl

**Supplementary Figures**

**Fig. B.1.** Summary of the risk of bias assessment.

**Fig. B.2.** Forest plots for the recovery outcomes.

**Fig. B.3.** Subgroup analyses for recovery outcomes according to flumazenil use.

**Fig. B.4.** Meta-regression for recovery outcomes according to flumazenil use.

**Fig. B.5.** Sensitivity analyses (leave-one-out technique) for all outcomes.

**Fig. B.6.** Funnel plots of all outcomes.

**Fig. B.7.** Trial-sequential analysis of the endpoint of hypotension, with power = 90%.

**Fig. B.8.** Trial sequential analyses for mean arterial pressure, heart rate, bispectral index (BIS), and bradycardia (power = 90%).

**Fig. B.9.** Trial sequential analyses for mean arterial pressure, heart rate, bispectral index (BIS), and bradycardia (power = 99%).

**Fig. B.1. Summary of the risk of bias assessment**


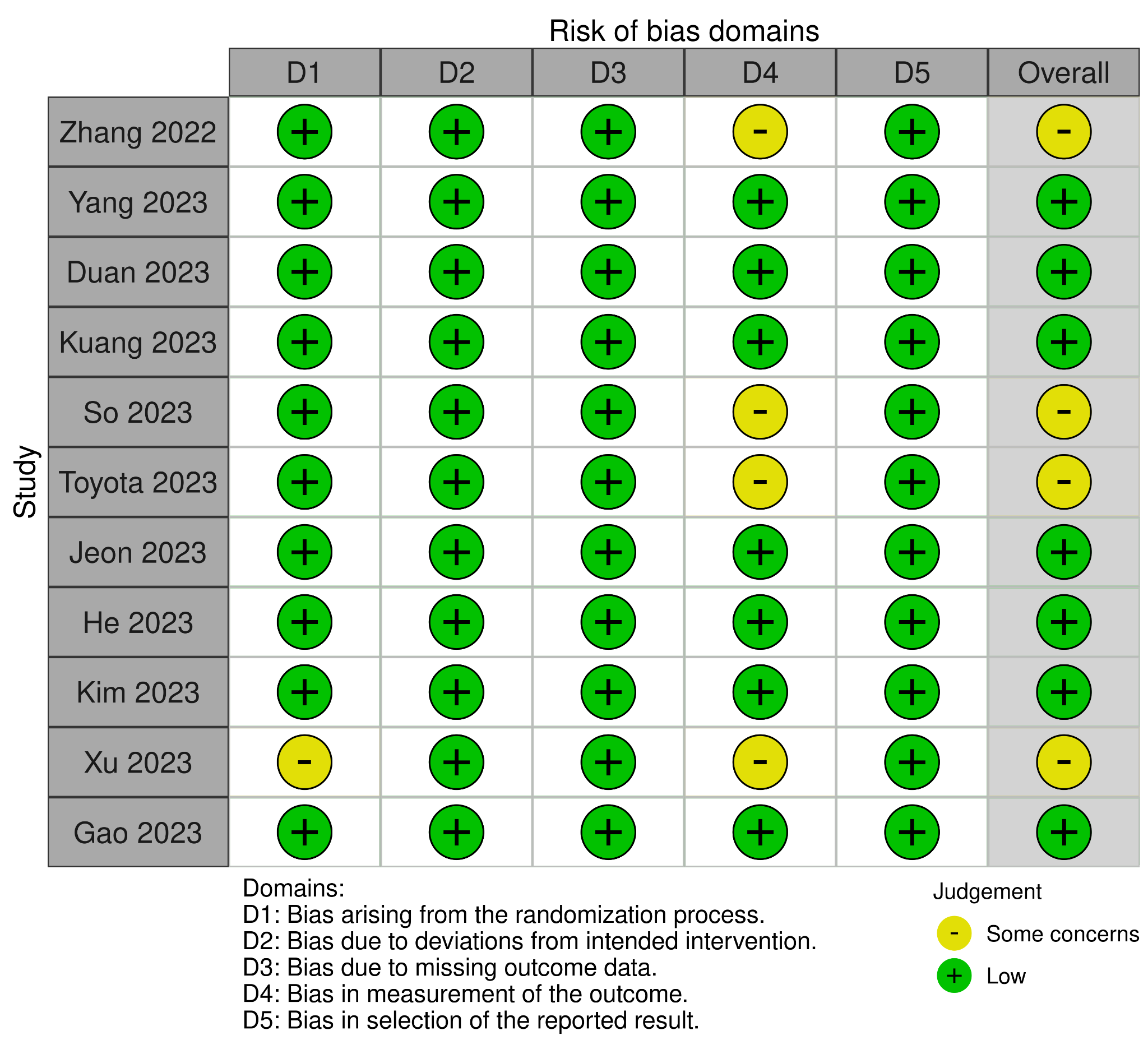


**Fig. B.2a**. There was no difference in emergence time between groups; **Fig. B.2b**. There was no difference in extubation time between groups; **Fig. B.2c**. There was no difference in the incidence of emergence agitation between groups.


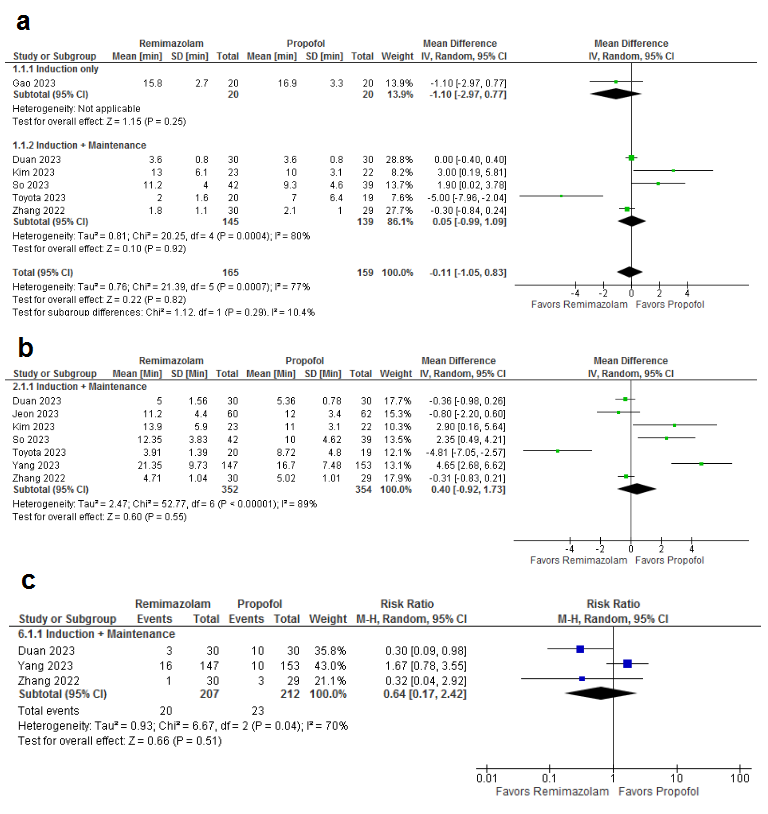


**Fig. B.3.** Subgroup analyses for the recovery outcomes according to flumazenil use. **Fig. B.3a**. In subgroup analysis according to flumazenil use, there was no difference in emergence time between groups; **Fig. B.3b**. In subgroup analysis according to flumazenil use, there was no difference in extubation time.


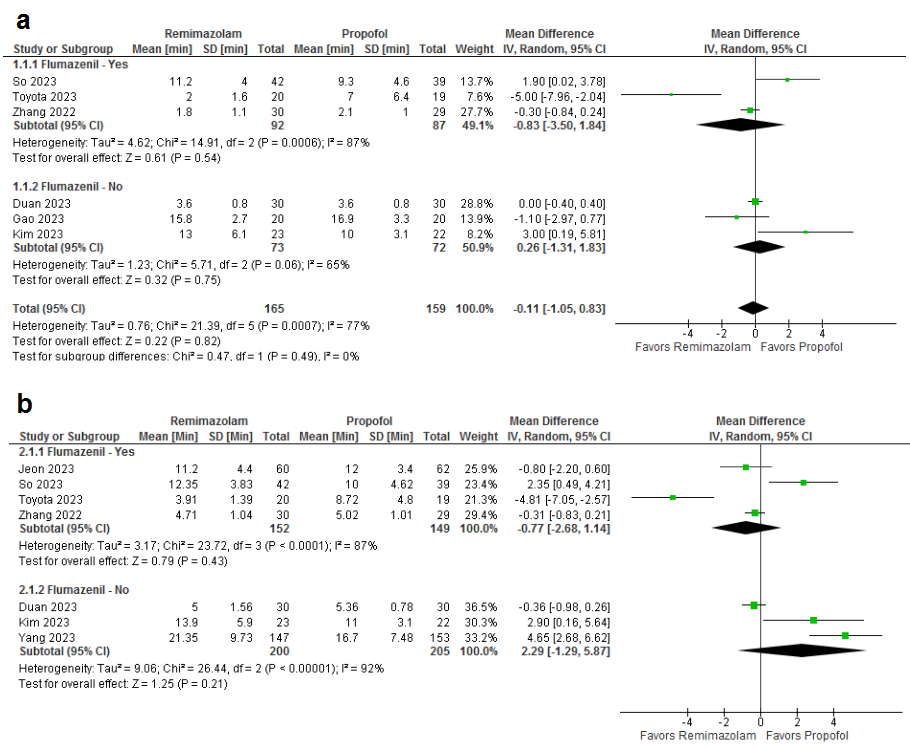


**Fig. B.4a**. Meta-regression for recovery outcomes according to flumazenil use on emergence time; **Fig. B.4b**. Meta-regression for recovery outcomes according to flumazenil use on extubation time.


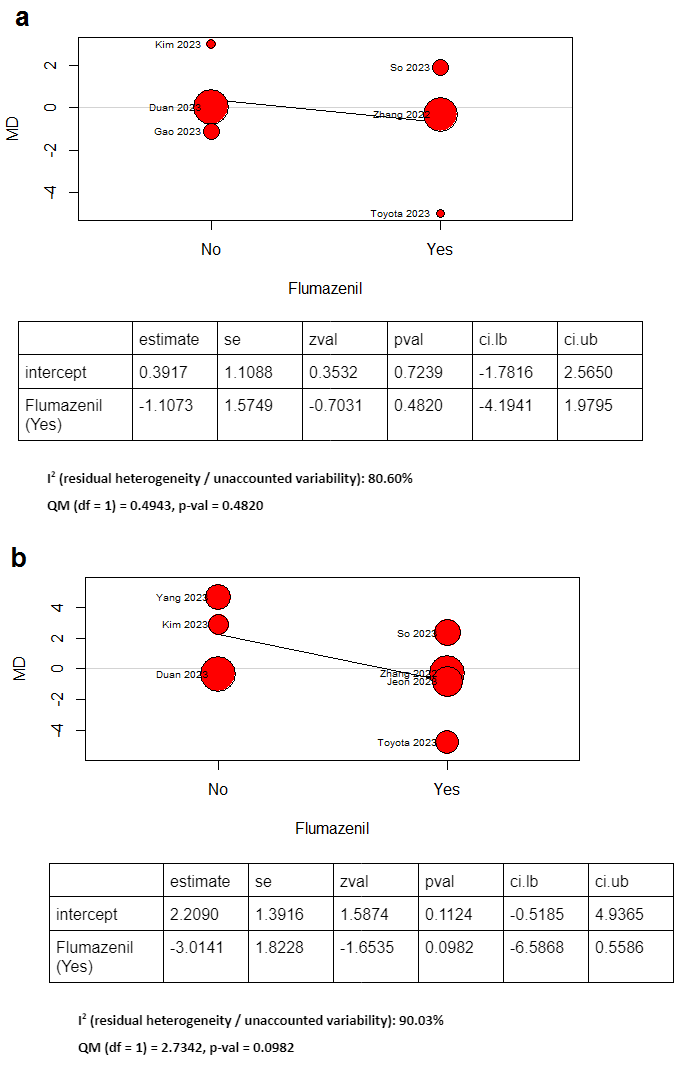


**Fig. B.5a**. - Leave-one-out sensitivity analysis on the incidence of hypotension; **Fig. B.5b**. Leave-one-out sensitivity analysis on the time to loss of consciousness; **Fig. B.5c**. Leave-one-out sensitivity analysis on the incidence of injection pain; **Fig. B.5d**. Leave-one-out sensitivity analysis on anaesthetic depth (BIS); **Fig. B.5e**. Leave-one-out sensitivity analysis on mean arterial pressure; **Fig. B.5f**. Leave-one-out sensitivity analysis on heart rate; **Fig. B.5g**. Leave-one-out sensitivity analysis on the incidence of bradycardia; **Fig. B.5h.** Leave-one-out sensitivity analysis on the incidence of emergence agitation; **Fig. B.5i**. Leave-one-out sensitivity analysis on emergence time; **Fig. B.5j.** Leave-one-out sensitivity analysis on extubation time.


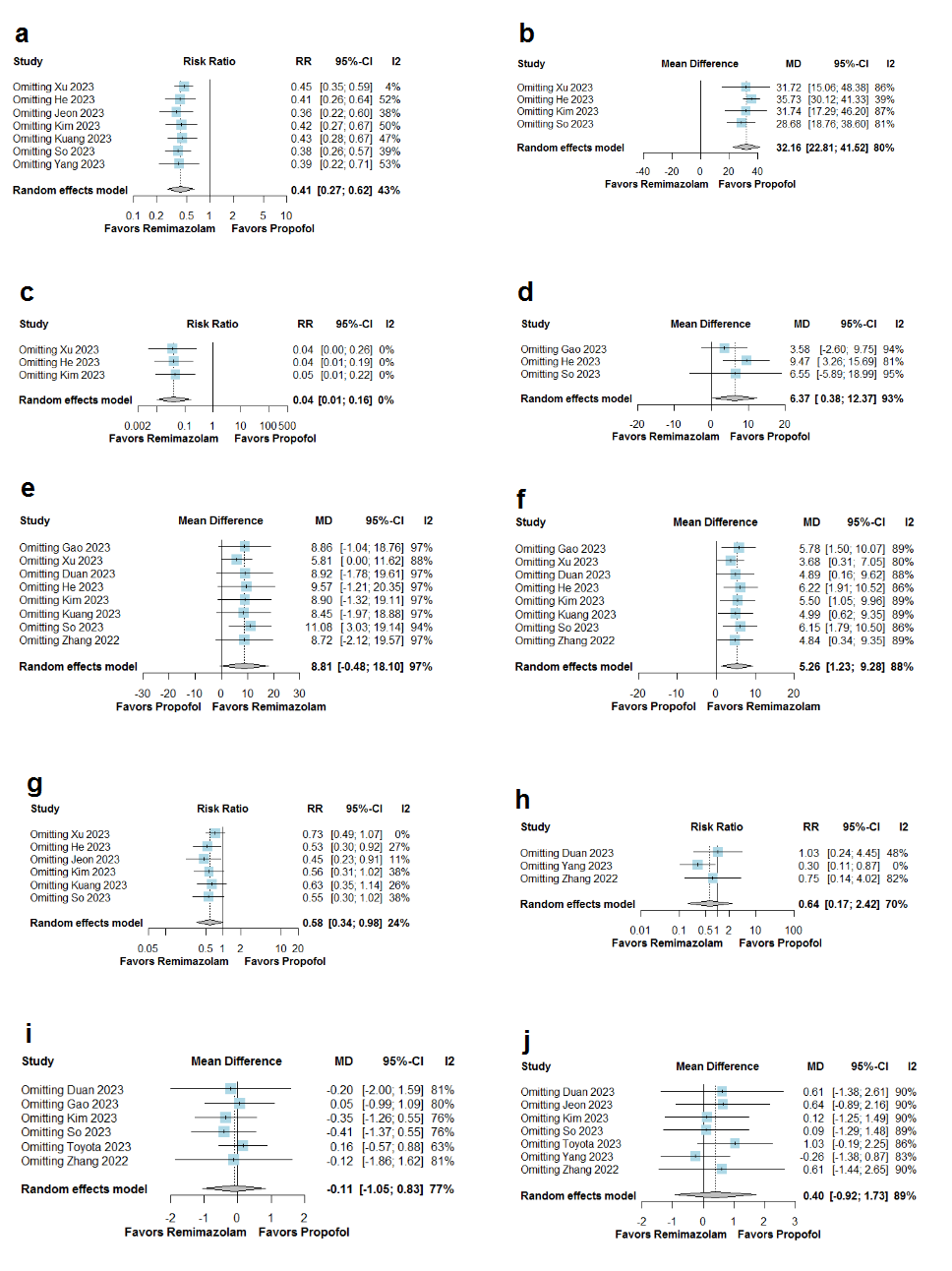


**Fig. B.6a.** Funnel plot on the incidence of hypotension; **Fig. B.6b.** Funnel plot on time to loss of consciousness; **Fig. B.6c.** Funnel plot on the incidence of injection pain; **Fig. B.6d.** Funnel plot on anaesthetic depth (BIS); **Fig. B.6e.** Funnel plot on mean arterial pressure; **Fig. B.6f.** Funnel plot on heart rate; **Fig. B.6g**. Funnel plot on the incidence of bradycardia; **Fig. B.6h.** Funnel plot on the incidence of emergence agitation; **Fig. B.6i.** Funnel plot on emergence time; **Fig. B.6j.** Funnel plot on extubation time.


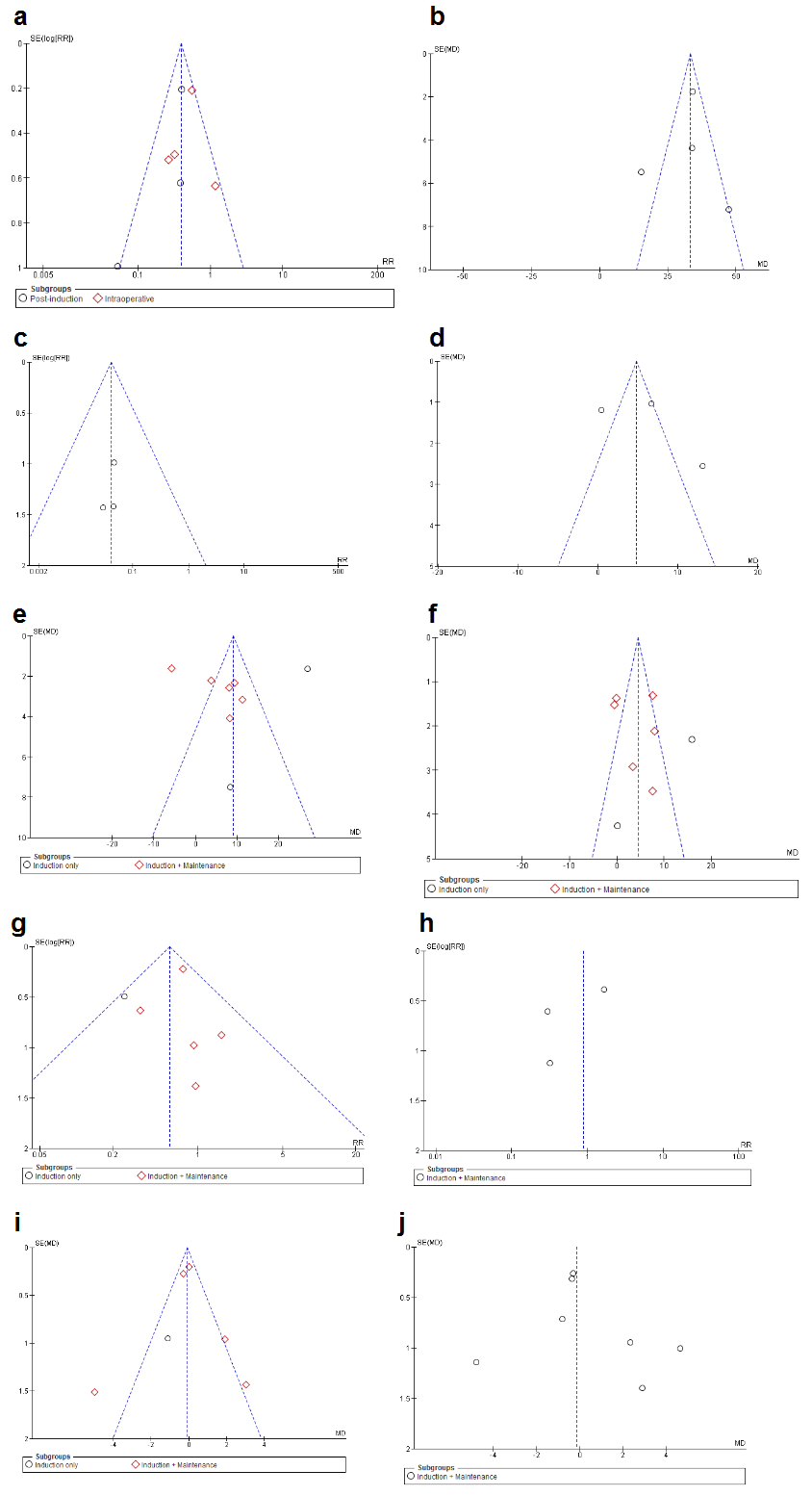
**Fig. B.7.** Trial-sequential analysis of the endpoint of hypotension, with power = 90%.


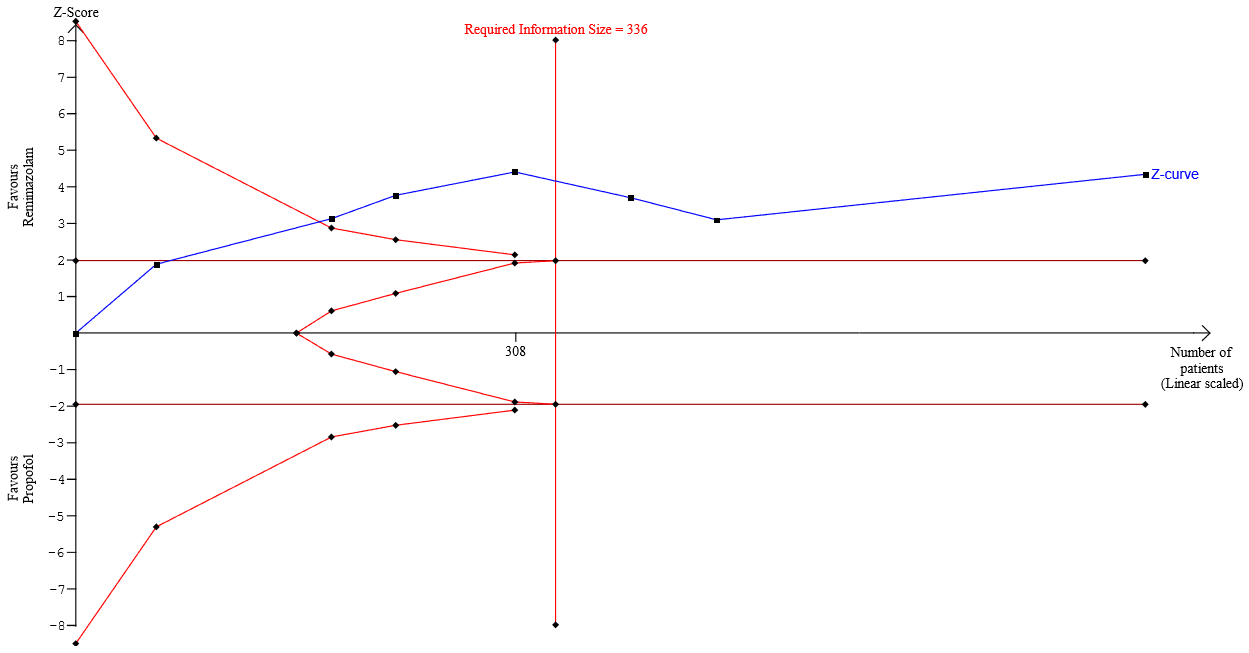


**Fig. B.8.** Trial sequential analyses with power = 90%. **Fig. B.8a.** Trial sequential analyses on the incidence of bradycardia; **Fig. B.8b.** Trial sequential analyses on anaesthetic depth (BIS); **Fig. B.8c.** Trial sequential analyses on mean arterial pressure; **Fig. B.8d.** Trial sequential analyses on heart rate.


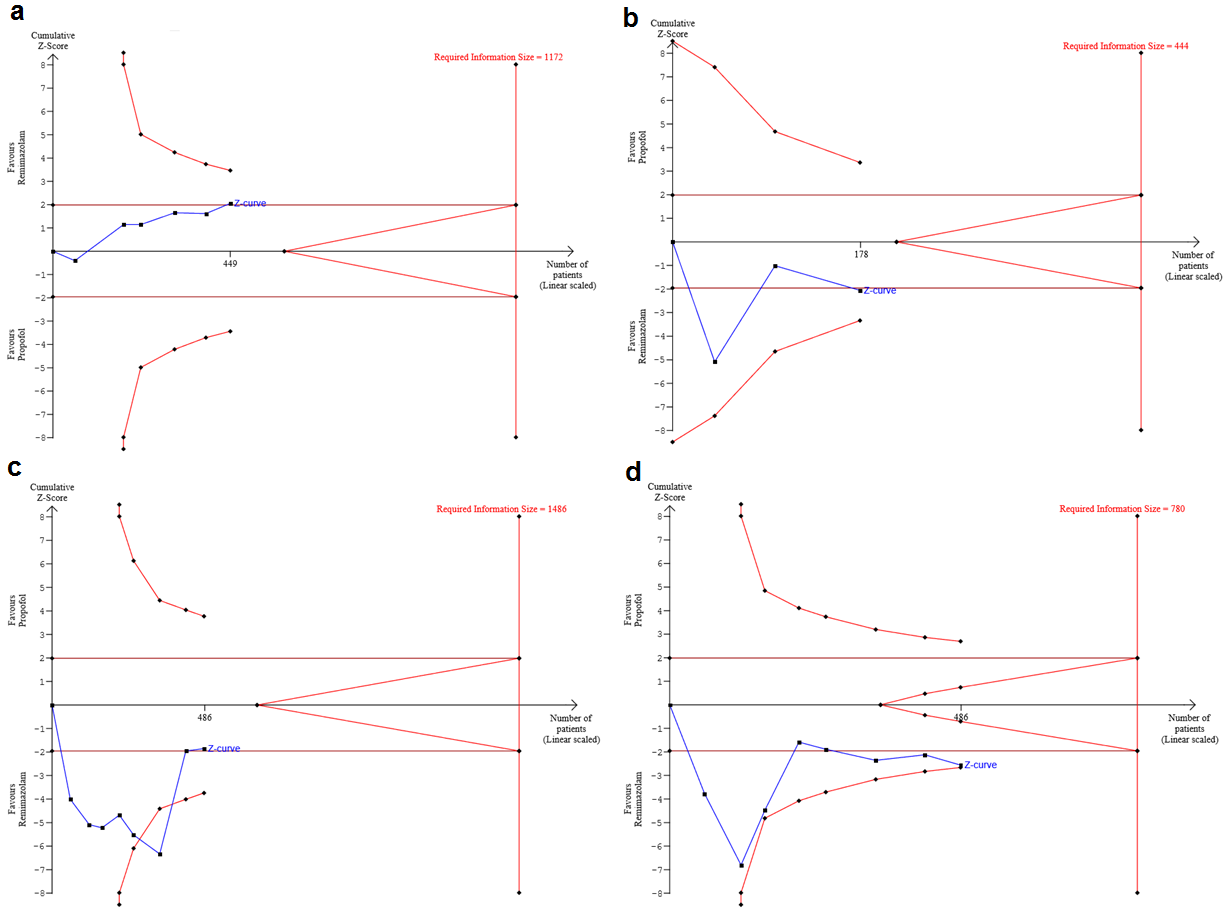


**Fig. B.9.** Trial sequential analyses with power = 99%. **Fig. B.9a.** Trial sequential analyses on the incidence of bradycardia; **Fig. B.9b.** Trial sequential analyses on anaesthetic depth (BIS); **Fig. B.9c.** Trial sequential analyses on mean arterial pressure; **Fig. B.9d.** Trial sequential analyses on heart rate.


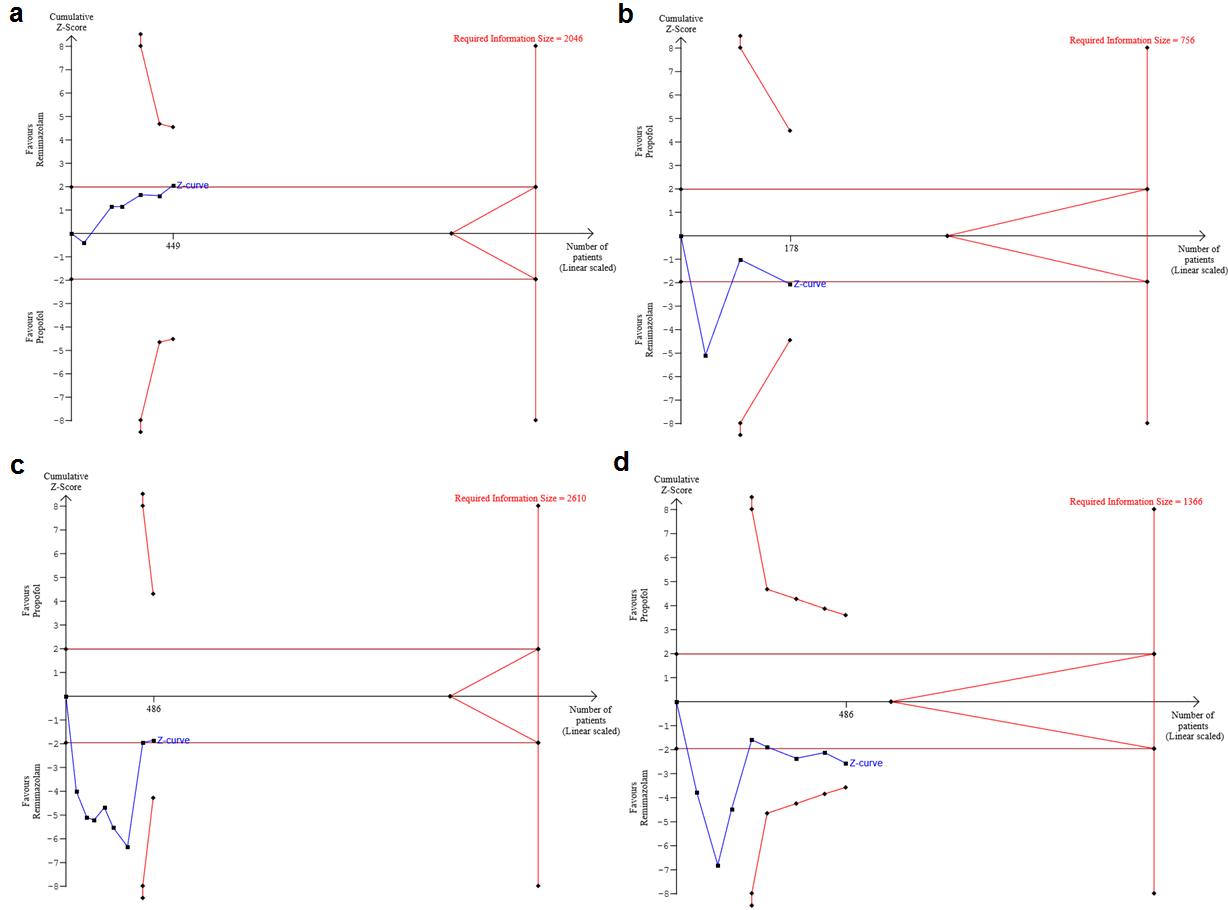

Supplement: Supplemental Digital Content [file ejanet-41-738-s002.docx]
